# Cross-cultural adaptation, reliability, validity and responsiveness of the Michigan Hand Outcomes Questionnaire (MHQ-Sp) in Spain

**DOI:** 10.1186/s13018-024-04723-x

**Published:** 2024-04-22

**Authors:** María Visitación Martínez-Fernández, Carmen María Sarabia-Cobo, Nuria Sánchez-Labraca

**Affiliations:** 1 Department of Rehabilitation, Mutua Montañesa Hospital, Santander, Cantabria Spain; 2 Department of Nursing, Universidad de Cantabria, Santander, Cantabria Spain; 3 IDIVAL- Health Research Institute Valdecilla, Santander, Cantabria Spain; 4 Department of Nursing, Physiotherapy and Medicine, Faculty of Health Sciences, University of Almería, Almería, Spain

**Keywords:** Reliability, Validity, Cross-cultural adaptation, Responsiveness, MHQ-Spain

## Abstract

**Background:**

The Michigan Hand Outcomes Questionnaire (MHQ) is a self-report tool widely recognized for measuring the health status of patients with hand and wrist problems from a multidimensional perspective**.** The aim of this study is to translate and culturally adapt the MHQ and validate its psychometric properties of validity, reliability, and responsiveness for different hand problems in Spain.

**Methods:**

The MHQ was translated and culturally adapted following the recommendations of the American Association of Orthopaedic Surgeons. The validation process adhered to the current Consensus-Based Standards for the Selection of Health Status Measurement Instruments (COSMIN) group and was conducted on 262 hand patients. Reliability was assessed through internal consistency using Cronbach's alpha. The study evaluated the test–retest reliability of the measurements using the intraclass correlation coefficient (ICC). Additionally, the measurement error was calculated using the standard error of measurement (SEM) and smallest detectable change (SDC). To assess the structural validity, confirmatory factor analysis (CFA) was employed, while construct validity was evaluated using Pearson's correlation coefficient. Finally, responsiveness was assessed using effect size (ES), standardized response mean (SRM), and minimum clinically important difference (MCID).

**Results:**

The reliability of the test was confirmed through internal consistency analysis, with a good Crombach's Alpha (0.82–0.85), and test–retest analysis, with good values of ICC (0.74–0.91). The measurement error was also assessed, with low values of SEM (1.70–4.67) and SDC (4.71–12.94)). The CFA confirmed the unidimensionality of each scale with goodness of fit indices, while the MHQ showed a high and negative correlation with DASH (*r* = − 0.75, *P* < 0.001) and DASH-work (*r* = − 0.63, *P* < 0.001) and was irrelevant with EQ-5D (*r* = − 0.01, *P* > 0.005) and grip strength (*r* = 0.05, *P* > 0.005). At week 5, all 222 patients across the three diagnosed hand subgroups showed moderate to high values above 0.92 for ES and SRM, with one MCID above 6.85.

**Conclusions:**

The MHQ-Sp was culturally adapted, and the results of this version showed good reliability and validity as well as high responsiveness for a wide range of hand conditions after surgical or conservative treatment in Spain.

**Supplementary Information:**

The online version contains supplementary material available at 10.1186/s13018-024-04723-x.

## Background

Wrist and hand are common areas of upper limb injury [1, 2]. In Spain in 2022, fractures of the hand accounted for 29.7% of upper limb fractures, 7.6% of fractures in trauma emergencies [3] and 21.85% of work injuries [4]. Faced with these high figures, healthcare professionals are challenged to measure the extent and impact of these effects [5, 6]. For this reason, patient-reported outcome measures (PROMs) have been developed from the patient's perspective and without the intervention of a healthcare professional [7–9]. Adding another dimension to the results of clinical evaluation or treatment effectiveness [7].

With this in mind, the Michigan Hand Outcomes Questionnaire (MHQ) was created at the University of Michigan in 1998 by Chung et al. [10]. The MHQ has been developed under rigorous psychometric principles as a multi-dimensional measure of the health status of patients with all types of hand and wrist impairments. This questionnaire assesses the right and left hand separately to avoid the dominance effect, differentiates between functional status and symptoms, and provides two unique scales such as aesthetics and satisfaction [11]. The MHQ, along with the Disability of Arm, Shoulder and Hand (DASH) [12], is the most widely used hand PROM [6]. Their validity, reliability and responsiveness have been demonstrated in a wide range of conditions including carpal tunnel syndrome (CTS) [13, 14], distal radius fractures [14, 15], osteoarthritis [16] and Dupuytren disease [17].

On the other hand, we must consider that the use of PROMs is related to the role of the hand in different cultures, which affects task performance and therefore scale responses and scores, as well as psychometric properties [18]. This means that a validated and adapted instrument is more accurate in clinical and research practice [18]. The MHQ has been officially translated and validated in 14 countries [11, 19–32].

There is currently a need for a specific outcome measure for hand and wrist injuries in Spain that is valid, reliable and able to detect clinical changes. Therefore, the general objective of this study is to create a Spanish version of the MHQ through a first process of cultural adaptation and a second process of validation of its psychometric properties.

## Methods

This descriptive, cross-sectional, psychometric validation study was conducted in a first stage of translation and cultural adaptation and a second stage of validity, reliability and responsiveness analysis. Permission was first requested and obtained from the authors with code MHQ IR code #3372.

All *participants* were randomly selected and previously diagnosed by a hand surgeon at the Mutua Montañesa Hospital in Santander, between January 2021 and September 2022. Inclusion criteria included patients aged between 18 and 65 years, of both genders, with acute trauma or neuromusculoskeletal involvement of the hand or wrist, and with sufficient Spanish to understand and complete the questionnaires. Exclusion criteria included patients with central nervous system problems, mental illness, behavioural disorders or involvement above the wrist. The recommended sample size was a minimum of 259 patients, following the principle of 4 to 10 patients per item for samples larger than 100 patients [33, 34].

Physiotherapy treatments included hydrotherapy, electrotherapy and manual therapy following surgical or conservative treatment.

*Outcome measures.* Sociodemographic data such as age, gender, dominant and affected hand were collected concurrently with baseline clinical measures. In order to ensure the response and participation rate, a continuous and personalised follow-up was carried out using self-administered and online electronic means, contacting patients in case of non-response, thus ensuring the total response rate. The following measures were used in this study:

Grip strength using the Baseline® Hydraulic Hand Dynamometer, which provides the average of the three measurements on each hand in a standardised seated position.

The MHQ [10] consists of 37 items assessing 6 domains: Overall hand function, activities of daily living (ADL), work performance, pain, aesthetics and satisfaction with hand function. It includes a Likert scale with response options from 1 to 5, with raw scores per domain converted to a range of 0 to 100 and the pain domain inverted. The total score is calculated as the sum of the six scores divided by six. The logarithm for its calculation is provided by the authors on page of the questionnaire [10, 35]. Higher scores indicate better function and, for the pain domain, greater severity. In this analysis, scores were recorded for the affected hand [10].

The DASH [12, 36], consists of a core module of 30 items measuring function and symptoms, as well as two optional 4-item modules that focus on music/sports and work. Each item consists of 5 response options, scored from 1 to 5. In the core module, the score ranges from 30 to 150 points, translated into a scale from 0 or no disability to 100 or more disability [12, 36]. The DASH-work module contains 5 items on a Likert scale from 1 to 5, all of which must be answered for a score to be calculated. It is scored from 0 to 100, with lower scores indicating better work ability.

European Quality of Live- 5 Dimensions (EQ-5D-3L) [37], is a generic instrument that includes in a first questionnaire five dimensions of health-related quality of life with three response options. Its calculation is based on a 5-digit number converted into a single index, with values recorded in Spain ranging from − 0.224 to 1 [38, 39], the lowest values indicating the worst health. For their use, permission and registration was requested from the authors via their website [40]. The EQ-5D includes a second section or "Visual Analogue Scale" (EQ-VAS) with scores from 0 to 100, ranging from worst to best health [41, 42].

Pain was measured using a visual analogue scale (VAS) on a scale of 0 to 100, where 0 is no pain and 100 is the most unbearable pain [43].

### Translation and cross‑cultural adaptation

This initial stage followed the steps recommended by the guidelines of the American Association of Orthopaedic Surgeons (AAOS) [18] (See Additional file 1).

Stage 1: A direct translation and summary was carried out by two native Spanish translators (a hand surgeon and a philologist) to obtain the first Spanish version of the MHQ (T1 and T2). The translators (T1 and T2) conducted their translations independently.

Stage 2: Synthesis of translations. Comparison of the two documents and consensus synthesis with a hand surgeon produced the version (T-12).

Stage 3: A back-translation from Spanish to English was then carried out by two native English translators (English teachers) who produced the BT-12 version.

Stage 4: Committee of experts. The team consisted of a methodologist, a philologist, two hand surgeons, and two translators who evaluated the idiomatic, semantic, experiential, and conceptual equivalences. The report's pre-final version was obtained. The semi-structured interview was conducted by the principal researcher of the study.

Stage 5: A pre-test or pilot study was conducted on a sample of 30–40 patients not included in the general sample [18, 44–46]. Content validity was then assessed by expert judgement by calculating Kendall's w concordance index, where 1 is perfect agreement and 0 is total disagreement[47]. Semi-structured interviews were conducted with patients to assess difficulty. Observations with more than 15% difficulty were considered for modification. The time taken to complete the questionnaire was recorded.

Stage 6: Finally, the final version of MHQ-Sp was produced and submitted with all reports to the authors for final approval (See Additional file 2).

### Psychometric testing of the MHQ-Sp

In this second part, the recommendations of the current Consensus-Based Standards for the Selection of Health Status Measurement Instruments (COSMIN) Group [48–50] were followed.

### Internal consistency

Internal consistency is the degree of interrelationship between items of the same measurement construct [51]. It was calculated using Cronbach's α index for the baseline scores, with values between 0 and 1, with ≥ 0.70 considered adequate, up to 0.9 good and above 0.9 redundant [52].

### Test–retest reliability

Test–retest reliability is the degree to which repeated measurements show similar results, based on the stability of patients on the construct [51]. It was estimated by the intraclass correlation coefficient (ICC) with values between 0 and 1, with > 0.70 considered as good reliability [52]. The MHQ was administered a second time at 10–15 days [53], without treatment, under the same conditions of administration and without prior knowledge of the previous measurements. The MHQ questionnaire was administered once the patient was diagnosed and before starting the first physiotherapy session.

### Meauremnet error

The measurement error expresses systematic or random errors in the scores that are not due to changes in the construct [51]. The standard error of measurement (SEM) with the formula: the difference between the test–retest per √2 and the smallest detectable change (SDC) with the formula: 1.96 × √2 × SEM was used [50, 54].

### Structural validity

Is defined as the degree to which the scores on the instrument reflect the dimensionality of the construct [51]. First, an exploratory factor analysis (EFA) was carried out using principal component analysis with Varimax orthogonal rotation. A confirmatory factor analysis (CFA) was then carried out to check whether the factor structure had correct goodness of fit indices, using the metrics: Tucker-Lewis Index (TLI) (0.95–1); Comparative Fit Index (CFI) (0.95–1); Standardized Root Mean Square Residual (SRMR) < 0.08; Root Mean Square Error of Approximation (RMSEA) < 0.06; Akaike Information Criterion (AIC); Expected Cross-Validation Index (ECVI); Chi-square (χ2) and chi-square divided by degrees of freedom χ2/gl (1.5–3) to assess the model fit [55].

### Construct validity

Is the relationship of an instrument's scores to other measures according to the theoretical hypothesis about the constructs being measured [52, 56]. In hypothesis testing, the instruments chosen for convergent validity, or measures with similar constructs [50], were DASH and DASH-work [12, 36] and for discriminant validity, or measures with different constructs [50] were EQ-5D [37] and grip strength, using Pearson's correlation coefficient. In accordance with the recommendations of the COSMIN group [48–50], three hypotheses have been proposed for convergent validity: (1) MHQ and DASH correlate highly and negatively, (2) MHQ-work correlates at least moderately and negatively with DASH-work, and (3) MHQ function correlates at least moderately with MHQ-ADL. For discriminant validity, two hypotheses were formulated: (1) the MHQ correlates weakly with grip strength and (2) the MHQ correlates weakly and negatively with the EQ-5D. For the size of the correlation, the following rule of thumb was used: low 0.30 < *r* < 0.50; medium 0.50 < *r* < 0.70 and high 0.70 < *r* < 0.90 [57].

### Responsiveness

Is the ability of a PROM to detect clinically important changes in the measured construct over time [52, 56]. Analysis was performed at 5 weeks post-treatment, starting with the weighting of changes between baseline and post-treatment scores by: descriptive analytical approach in box plots for subgroups, t-student for average of differences, effect size (ES) calculated by: mean change/DE baseline measurement and standardised response mean (SRM): mean change/DE change. The ES and SRM values reflect sensitivity to change with 0.20 indicating low, 0.50 moderate and 0.80 high [58, 59]. The minimun clinically important difference (MCID) was then calculated to indicate the effectiveness of physiotherapy in the three subgroups. The anchor method was used by observing the sample at one point in time and grouping patients into categories according to external criteria of satisfaction [60]. According to the COSMIN group recommendations [51], the hypotheses were: (1) improvement in nerve injury patients would be less than in radius fracture patients, and (2) the ES of MHQ-work and DASH-work would be equivalent.

Finally, the interpretability [51] was assessed by the area under the curve (AUC) using the receiver operating characteristic (ROC) curve to discriminate between different levels of function. AUC values range from 1 to 0.5, indicating better to worse discriminatory ability [61].

**Fig. 1:**
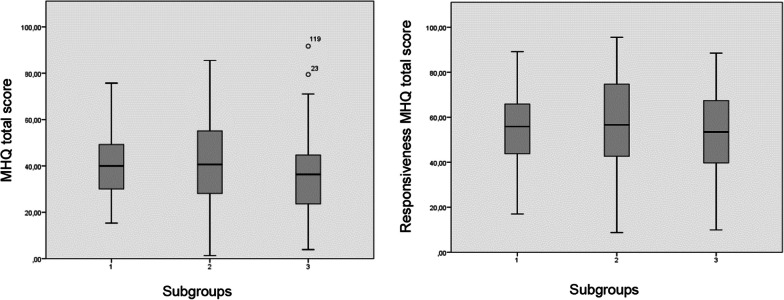
Responsiveness box plots before and after the intervention in the three diagnostic subgroups

**Fig. 2:**
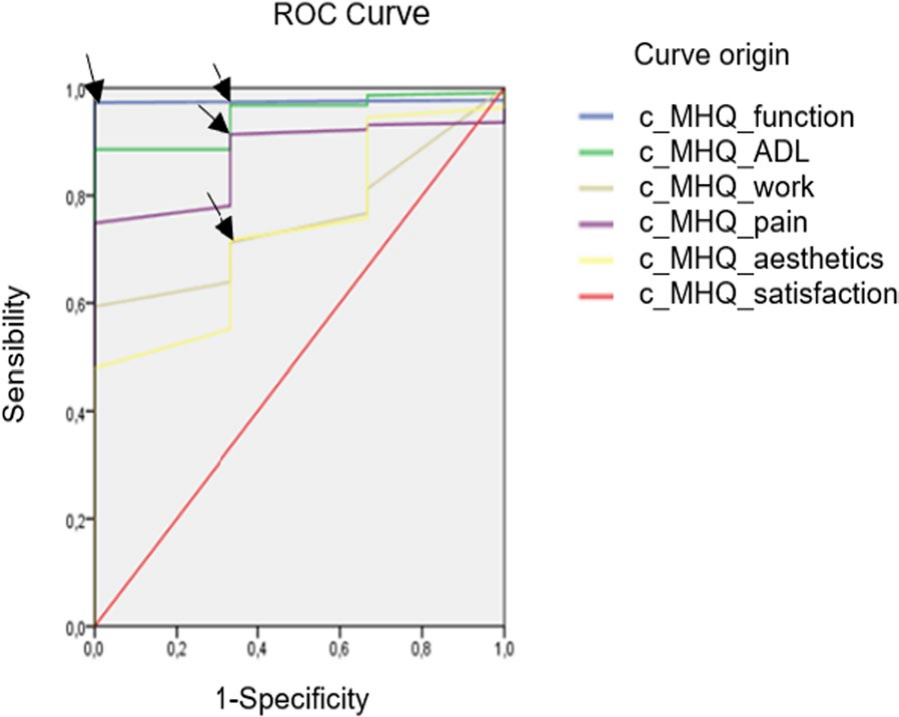
ROC curve for interpretability

*Statistical análisis.* Means and standard deviations (SD) were used for quantitative variables and frequencies and percentages for categorical variables. IBM SPSS Statistics 25.0 was used to calculate psychometric properties, ROC curve and box plots (Figs. 1, 2) and SPSS Amos 20 was used for factor analysis. The sample size was calculated according to the recommendations of Terwee et al. [52] and Vet et al. [34] of 7 patients per item and samples larger than 100 patients.

## Results

The sample of patients who completed all items of the questionnaires was 262 patients with various musculoskeletal conditions of the hand or wrist, out of 286 invited to participate (Table 1). (24 out of 286 patients were excluded from the study because they did not return the questionnaire once it was administered or they returned it unanswered or their treatment was changed after administration).

**Table 1:** Demographic data of patients (n = 262)

Variable	Mean ± SD	N	%
*Age*			
Male	45 ± 10		
Female	50 ± 11		
*Sex*			
Male		167	63.74
Female		95	36.26
*Dominant hand*			
Right		231	88.16
Left		19	7.25
Both		12	4.58
*Injured hand*			
Left		109	41.60
Right		137	52.29
Both		16	6.10
*Diagnosis*			
Distal radial + ulnar fractures		7	2.67
Distal ulnar fracture		2	0.76
Distal radial fracture		39	14.88
Phalangeal amputation		10	3.81
Rhizarthrosis		6	2.29
Tendon injuries		30	11.45
CTS^a^		19	7.25
TFCC^b^		6	2.29
Finger sprains		13	4.96
Ligament injuries		20	7.63
Tendinophaties		20	7.63
Phalangeal/metacarpal fractures		30	11.45
Carpal injuries/ fractures		17	6.48
Wounds/cuts/lacerations		16	6.10
Others		27	10.30

^a^*CTS* carpal tunnel syndrome

^b^*TFCC* triangular fibrocartilague complex

Of the 262 patients, 145 (55.34%) received surgery and physiotherapy, 105 (40.07%) received conservative treatment with physiotherapy, and 12 (4.59%) received other conservative treatments such as immobilization or medication. Physiotherapy treatments included hydrotherapy, electrotherapy and manual therapy following surgical or conservative treatment.

### Translation and cross-cultural adaptation

Translation and back-translation processes were carried out during the preparation of their reports. The changes were minor and agreed by consensus and concerned the response options in the function domain:—In the 1st domain Function: Within the options of this domain the adjectives "fair, poor or very poor" translated as "fair, scarce or very scarce", the adverbs "regular, bad or very bad" were chosen, being more appropriate to the question.

In the 4th domain Pain: In the 3rd question of the pain domain "interfere" was replaced by "caused alterations" and in the 5th question the adjective "unhappy" was replaced by "negatively affected his mood”.

The pre-final version was used in the pilot study with a sample of 33 patients, in which content validity was performed with an inter-expert agreement of Kendall's (w = 0.8, *p* < 0.001). The final version and all reports were sent to the authors for approval by the Michigan Center for Hand Outcomes and Innovation Research. Difficulty was less than 15% and the average time taken to complete the MHQ was 12 min.

### Validation of psychometric properties

#### Internal consistency, test–retest reliability and measurement error

Internal consistency was calculated using the baseline scores of the 262 patients with adequate Cronbach's alpha values ranging from 0.821 to 0.858, which did not improve when we eliminated any of the domains. Test–retest reliability was assessed on a sample of 64 patients from the general sample who completed the MHQ for the second time. Good reliability was obtained with ICC values ranging from 0.74 to 0.91. In the measurement error analysis, the SEM was 1.8 and the SDC was 4.99 for the total MHQ score, indicating a tendency towards consistency for the individual scores and relatively little effect of measuring error (Table 2).

**Table 2:** Internal consistency, test–retest reliability, measurement error and floor/ceiling effects of MHQ

Instrument	Basal Mean ± SD	Post-test Mean ± SD	95% Confidence interval		ICC	SEM	SDC	Cronbach’s α	Floor effect %	Ceiling effect %
			Lower	Upper						
MHQ function	43.52 ± 17.95	42.46 ± 20.50	0.70	0.86	0.78	2	5.54	0.82	8	0.8
MHQ ADL	34.97 ± 25.26	28.97 ± 18.92	0.69	0.82	0.74	2.40	6.65	0.82	5	0.8
MHQ work	24.68 ± 26.74	23.13 ± 23.78	0.74	0.92	0.84	4.67	12.94	0.85	32.8	1.9
MHQ pain	43.72 ± 21.06	43.13 ± 20.32	0.87	0.96	0.91	4.20	11.64	0.85	4.6	0
MHQ aesthetics	57.03 ± 27.39	55.86 ± 28.01	0.71	0.94	0.84	2.90	8.04	0.85	2.7	12.6
MHQ satisfacion	35.56 ± 22.93	33.33 ± 20.10	0.70	0.90	0.81	1.70	4.71	0.82	3.8	0.8
MHQ total score^a^	39.92 ± 16.71	37.81 ± 15.67	0.79	0.91	0.87	1.80	4.99	0.85	0.4	0

^a^Sum of all domains divided by 6

### Structural validity

It was carried out on the original sample of 262 patients and the CFA showed a good fit to the original model. The dimensionality of each domain was confirmed by: TLI = 0.93 IC 95% (0.90–1.00); CFI = 0.97 IC 95% (0.90–1.00); SRMR = 0.06 IC 95% (0.02–0.10); RMSEA = 0.04 IC 95% (0.01–0.08); AIC = 108.20; ECVI = 0.22; χ2 = 81.27 y χ2/gl = 2.35.

### Construct validity

The hypotheses of convergent validity were confirmed: (1) MHQ correlated highly with DASH (*r* = − 0.75, *p* < 0.001); (2) MHQ-work and DASH-work correlated moderately (*r* = − 0.64, *p* < 0.001) and (3) MHQ-function and MHQ-ADL correlated moderately (*r* = 0.66, *p* < 0.001). For divergent validity, both hypotheses were confirmed as for EQ-5D (*r* = − 0.01, *p* < 0.001) and for grip strength (*r* = 0.05, *p* < 0.001). The correlation with MHQ was irrelevant, without statistical significance. On the other hand, high to moderate correlations of the MHQ with the other domains and with DASH and DASH-work (*r* = 0.61 to *r* = 0.79, *p* < 0.001) and irrelevant correlations with EQ-5D, EQ-VAS and grip (*r* = 0.001, *p* < 0.001) to (*r* = 0.13, *p* < 0.001) were observed (Table 3).

**Table 3:** Construct validity: correlations between MHQ and MHQ domains with DASH, EQ-5D, EQ-VAS, and Grip strength (N = 262)

Instrument	MHQ function	MHQ ADL	MHQ work	MHQ pain	MHQ aesthetics	MHQ satisfaction	MHQ total score	DASH	DASH work	EQ-5D	EQ-VAS
MHQ function	1										
MHQ ADL	0.66**^b^	1									
MHQ work	0.41**	0.46**	1								
MHQ pain	0.34**	0.36**	0.25**	1							
MHQ aesthetics	0.37**	0.35**	0.25**	0.32**	1						
MHQ satisfaction	0.61**	0.56**	0.38**	0.42**	0.37**	1					
MHQ total score^a^	0.77**	0.79**	0.66**	0.61**	0.65**	0.77**	1				
DASH	− 0.59**	− 0.76**	− 0.51**	− 0.46**	− 0.36**	− 0.52**	− 0.75**§	1			
DASH work	− 0.48**	− 0.55**	− 0.6**^b^	− 0.31**	− 0.28**	− 0.39**	− 0.63**	0.63**	1		
EQ-5D	− 0.05	− 0.03	0.04	0.08	− 0.05	− 0.04	− 0.01^c^	− 0.05	0.00	1	
EQ-VAS	− 0.00	0.04	− 0.06	0.00	− 0.05	− 0.00	− 0.02	0.02	0.00	− 0.06	1
Grip	0.04	0.02	0.13*	− 0.04	0.07	− 0.02	0.05^c^	− 0.06	− 0.04	0.12*	− 0.60**

^a^Sum of all domains divided by 6

^b^Correlation is significant for hypothesis test p ≤ 0.05

^c^Correlation is not significant for hypothesis test p ≥ 0.05

^**^Correlation is significant at the 0.01 level (2-tailed)

^*^Correlation is significant at the 0.05 level (2-tailed)

### Responsiveness

From the initial sample, 222 patients completed the MHQ, DASH and DASH-work as well as grip strength and VAS pain at 5 weeks after baseline. The difference between the subgroups was taken into account and represented: (Group1) wrist fractures (72 patients: 32.43%); (Group 2) nerve, tendon and soft tissue hand injuries (102 patients: 45.94%) and (Group 3) bone fractures in the hand and fingers and other hand injuries (48 patients: 21.62%). Firstly, an analytical approach was taken using descriptive box plots of the three subgroups (Fig. 1).

For the set of measures, the results were calculated and presented inferentially using the Student's t-statistic, the mean of the differences being (12.76 ± 15.39, *p* < 0.001). In the MHQ domains ES recorded values from 0.81 to 0.31 and SRM from 0.87 to 0.37, while in the total MHQ they were 0.75 and 0.82 respectively, indicating moderate to high responsiveness (Table 4). The values of ES and SRM in each subgroup showed a greater magnitude for the group of finger and hand injuries. Additionally, the ES values in DASH-work and MHQ-work were equivalent, confirming both hypotheses (Table 5).

**Table 4:** Responsiveness, of MHQ, DASH, DASH-work, Grip strenght and VAS-pain (N = 222)

Instrument	Mean ± SD before intervention	Mean ± SD after intervention	Mean difference ± SD	p	ES	SRM	SEM
MHQ function	43.25 ± 18.28	57.24 ± 18.83	13.98 ± 16.05	0.000	0.76	0.87	1.07
MHQ ADL	34.97 ± 25.26	55.52 ± 26.45	20.55 ± 24.17	0.000	0.81	0.85	1.62
MHQ work	24.67 ± 26.73	39.90 ± 30.66	15.22 ± 28.39	0.000	0.56	0.53	1.90
MHQ pain	43.72 ± 21.06	53.33 ± 24.27	9.61 ± 25.57	0.000	0.45	0.37	1.71
MHQ aesthetics	57.02 ± 27.39	65.62 ± 27.99	8.60 ± 22.52	0.000	0.31	0.38	1.51
MHQ satisfaction	35.55 ± 22.92	65.62 ± 27.99	14.93 ± 23.63	0.000	0.65	0.63	1.58
MHQ total score^a^	39.89 ± 16.91	52.66 ± 18.83	12.76 ± 15.39	0.000	0.75	0.82	1.03
DASH	52.39 ± 21.62	32.05 ± 19.98	− 20.19 ± 18.76	0.000	− 0.93	− 1.07	1.25
DASH work	73.93 ± 26.24	55.12 ± 27.02	− 18.55 ± 20.09	0.000	− 0.70	− 0.92	1.34
Grip	13.31 ± 10.02	16.35 ± 10.50	3.03 ± 11.25	0.000	0.30	0.26	0.75
VAS pain	4.78 ± 2.52	3.52 ± 2.41	− 1.26 ± 2.97	0.000	− 0.50	− 0.42	0.19

^a^Sum of all domains divided by 6

**Table 5:** Responsiveness. Mean and standard deviation (SD), standardized response mean (SRM) and confidence interval (CI) and effect size (ES) for subgroups by diagnosis

Treatment n (%)	Mean difference ± SD	Standardized response mean (SRM) (± CI)^a^	Effect size (ES)
Group 1	13.74 ± 3.44	0.77 (0.45)	0.95
Group 2	16.06 ± 3.79	0.75 (0.75)	0.92
Group 3	16.35 ± 2.07	0.85 (0.85)	0.96

^a^*CI* confidence Interval

In terms of MCID results, it was observed that patients with hand and finger injuries made the most progress (Table 6). In the interpretability analysis, using the satisfaction domain as a reference, AUC values of 0.75 to 0.97 were obtained, indicating a high ability of the MHQ to discriminate between patients who improved and those who did not (Table 6) (Fig. 2).

**Table 6:** Interpretability. AUC of the MHQ discriminative ability for the total group and MCID for three subgroups

Total group				Group 1	Group 2	Group 3
Domain	AUC	Sensitivity	1-Specificity	MCID	MCID	MCID
MHQ function	0.97	1	0	40.91	46.6	55.40
MHQ ADL	0.94	0.98	0.33	44.59	46.96	55.45
MHQ work	0.75	0.71	0.32	28.65	26.43	38.39
MHQ pain	0.87	0.91	0.34	52.60	25.65	48.04
MHQ aesthetics	0.73	0.71	0.32	6.85	34.52	57.76

## Discussion

The findings of our study demonstrate that the MHQ has a satisfactory level of cultural adaptation and strong evidence of validity, reliability, and responsiveness across a broad spectrum of hand and wrist injuries in Spain. The primary aim of this study was to traslate and adapt the MHQ into Spanish. The process did not require significant alterations from the original version [10]. No items were added or deleted. There was a modification by the authors since 1998, because when we were granted permission they sent us a version of the MHQ for the pain domain, consisting of 5 items for the right hand and 5 for the left hand instead of 5 general items. This does not affect the results as only one of the hands, the affected one, is chosen. There are 62 questions taking into account right and left hands, although it is considered a 37-item questionnaire.

The method employed was that developed by Beaton et al.[18], consistent with other adapted versions of the MHQ [11, 19, 20, 22–26, 28–31, 62–64]. According to the expert assessment, Kendall's 0.85 w values indicated a high coefficient of concordance, whereas two other versions [25, 26] considered an Item Objective Congruence (IOC) of 0.5 to be acceptable [65]. 

Internal consistency in reliability was good [66], and the Crombach's alpha values ranged from 0.82 a 0.85 in the pain domain, matching with three versions of the MHQ [19, 31, 67]. These values were lower than the original MHQ [10] (0.86–0.97) or other versions of DASH > 0.90 [68, 69]. The findings suggest a satisfactory correlation between the items while avoiding redundancy compared to other versions [26, 29, 64, 70]. It is important to note that values above 0.90 are common in large instruments, with this index being sensitive to the nunber of ítems [68].

Moderate results in ADL and good results in the other domains were found when test–retest reliability was examined, exceeding 0.78. Notably, the pain domain displayed an excellent value of 0.91, matching the original MHQ [10] and the Turkish version [19]. These favourable outcomes are linked to a sufficiently sample size, as the ICC is responsive to this information, indicating tangible transformations in health status for hand patients.[14, 68].

The SDC values were low, indicating low variability between measurements and consequently higher accuracy in the MHQ, ranging from 4.71 to 12.94, while the SEM ranged from 1.7 to 4.67. It is important to note that similar SEM values for the MHQ have been observed in patients with osteoarthritis [21], in conservative treatments and in a variety of hand injuries [24, 26].

MHQ's structure was assessed and found to be sound based on the CFI and TLI indices, indicating a good [71] and acceptable model fit [72]. Moreover, the indices scored over 0.95 and 0.90, respectively, reflecting the model's high fit. Likewise, both RMSEA and SRMR have values below 0.06 and 0.08, respectively [71]. These findings indicate comparable outcomes with the Thai version [26] and superior outcomes in comparison to the shortened version of Chung and Morris [73] or the Finnish version of the MHQ [32].

The COSMIN guide evaluates hypothesis testing based on studies, groups of 50 or more people, and at least 75% of the results being in agreement [52, 74]. The hypotheses were validated as MHQ and DASH displayed significant correlations of − 0.75, in line with different versions of MHQ (− 0.72 to − 0.84)[21–23, 26, 27, 29]. MHQ-work with DASH-work confirmed a moderate correlation (− 0.64) in accordance with the Polish version of MHQ [24]. The correlation between MHQ-function and MHQ-ADL was moderate (− 0.66), with comparable findings to the German version (0.54) [75]. Discriminant validity was assessed for both hypotheses, however, it did not achieve statistical significance. In the case of grip strength, this may be due to the heterogeneity of hand pathologies, which may show differences in the strength of the different muscle groups involved.

Effect sizes were high across all three subgroups (0.92 to 0.96), indicating greater efficacy of physiotherapy treatment, particularly for hand and finger conditions and bone fractures. Our data shows mean effect sizes similar to those reported by DASH (0.7) for patients with upper limb impairments [36, 69] or general hand problems [76]. The MCID calculation showed that the results matched the ones obtained by ES and SRM for the three groups of conditions. Specifically, the group of injuries and bone fractures of the fingers and hand, showed the most significant improvements after physiotherapy. The MCID values in all three subgroups were higher than MHQ values in patients with chronic conditions such as rheumatoid arthritis or CTS [14] and osteoarthritis [21] or various hand conditions [77].

This study has some limitations, such as treating acute injuries in a single centre and not being able to generalise its results to other types of hand problems, such as rheumatoid or neurological. Although applied physiotherapy treatments use common modalities in terms of hydrotherapy, electrotherapy and manual therapy, there is diversity in the procedures and methods of application. In this sense, it was not possible to approach this study from the perspective of treatment effectiveness by focusing on diagnosis by anatomical region. In addition, ROC curves could not be calculated for each subgroup because of the high satisfaction scores for the overall group.

## Conclusions

This study involved the translation and cultural adaptation of the MHQ, as well as the validation of its psychometric properties, for spanish hand patients who had received conservative or surgical treatment prior to physiotherapy. The results obtained indicate that MHQ-Sp is an instrument with good validity and reliability, as well as high responsiveness in all domains and for a broad group of hand injuries. Therefore, based on the results obtained, we recommend the MHQ as a multidimensional tool to assess the health and functionality of all types of acute traumatic and neuromusculoskeletal hand and wrist injuries in Spain.

### Supplementary Information

**Additional file 1:** Cross-cultural process used for MHQ-Sp.

**Additional file 2:** Michigan Hand Questionnaire in Spanish/ Cuestionario de evaluación de mano Michigan.

## Data Availability

The datasets analyzed during the current study are available from the corresponding author on reasonable request.

## Abbreviations

AAOS: American Association of Orthopaedic Surgeons
ADL: Activities of daily living
AIC: Akaike Information Criterion
AUC: Area Under the Curve
CFA: Confirmatory Factor Analysis
CFI: Comparative Fit Index
CI: Confidence Interval
COSMIN: Consensus-Based Standards for the Selection of Health Status Measurement Instruments
CTS: Carpal tunnel síndrome
DASH: Disability of Arm, Shoulder and Hand
ECVI: Expected Cross-Validation Index
EFA: Exploratory Factor Analysis
EQ-5D-3L: European Quality of Live-5 Dimensions
EQ-VAS: EQ-Visual Analogue Scale
ES: Effect size
ICC: Intraclass correlation coefficient
IOC: Item Objective Congruence
MCID: Minimun clinically important difference
MHQ: Michigan Hand Outcomes Questionnaire
PROMs: Patient-reported outcome measures
RMSEA: Root Mean Square Error of Approximation
ROC: Receiver operating characteristic
SD: Standard Deviation
SDC: Smallest detectable change
SEM: Standard error of measurement
SRM: Standardised response mean
SRMR: Standardized Root Mean Square Residual
TLI: Tucker–Lewis Index
