# Supplementary material for: Cross-cultural adaptation, reliability, validity and responsiveness of the Michigan Hand Outcomes Questionnaire (MHQ-Sp) in Spain
Source: J Orthop Surg Res. 2024 Apr 22;19:256. doi: 10.1186/s13018-024-04723-x (PMC11034153; doi:10.1186/s13018-024-04723-x)
Supplement: Supplementary file 1 — Additional file 1: Cross-cultural process used for MHQ-Sp. [file 13018_2024_4723_MOESM1_ESM.docx]

Additional file 1**.**
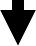

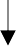
Cross-cultural process used for MHQ-Sp recommended by the American Association of Orthopaedic Surgeons (AAOS).

4 equivalences

Pretest. Pilot study n= 30-40

Back-translator1

Back-translator2

Consensus document

**Michigan Hand outcomes Questionnaire (MHQ). Original version**


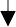

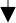


Translator2

StageVI. Submission and appraisal of all written reports by developers/committee.

**Spanish MHQ. Final document translated**

Stage V Pretesting. Report

Stage VI Expert committee.

Report

Stage III Back translation

Report

**Reverse translation**

Stage II Synthesis Report

**Comparation both document**

Stage I Direct translation

Report

Translator1
