# Supplementary material for: Cross-cultural adaptation, reliability, validity and responsiveness of the Michigan Hand Outcomes Questionnaire (MHQ-Sp) in Spain
Source: J Orthop Surg Res. 2024 Apr 22;19:256. doi: 10.1186/s13018-024-04723-x (PMC11034153; doi:10.1186/s13018-024-04723-x)
Supplement: Supplementary file 2 — Additional file 2: Michigan Hand Questionnaire in Spanish/ Cuestionario de evaluación de mano Michigan. [file 13018_2024_4723_MOESM2_ESM.docx]

Additional file 2

Cuestionario de evaluación de mano Michigan

**Instrucciones:** Este cuestionario pretende reflejar la percepción que tiene sobre sus manos y su estado de salud. Dicha información ayudará a llevar a cabo un seguimiento de cómo se siente y de la capacidad con la que puede realizar sus actividades habituales. Conteste *cada* pregunta marcando la respuesta indicada. En caso de duda, por favor elija la respuesta que más se adecúe.

1. Las siguientes preguntas se refieren a la función de su(s) mano(s)/muñeca(s) **durante la semana pasada** (Por favor, rodee con un círculo 1 respuesta para cada pregunta).
   1. Las siguientes preguntas se refieren a su mano/muñeca **derecha**

|  | Muy bien | Bien | Regular | Mal | Muy mal |
| --- | --- | --- | --- | --- | --- |
| 1. En general, ¿qué tal funcionó su mano **derecha**? | 1 | 2 | 3 | 4 | 5 |
| 2. ¿Qué tal se movieron sus dedos de la mano **derecha**? | 1 | 2 | 3 | 4 | 5 |
| 3. ¿Qué tal se movió su muñeca **derecha**? | 1 | 2 | 3 | 4 | 5 |
| 4. ¿Cómo fue la fuerza en su mano **derecha**? | 1 | 2 | 3 | 4 | 5 |
| 5. ¿Cómo describiría el tacto de su mano **derecha**? | 1 | 2 | 3 | 4 | 5 |

- 1. Las siguientes preguntas se refieren a su mano/muñeca **izquierda**

|  | Muy bien | Bien | Regular | Mal | Muy mal |
| --- | --- | --- | --- | --- | --- |
| 1. En general, ¿qué tal funcionó su mano **izquierda**? | 1 | 2 | 3 | 4 | 5 |
| 2. ¿Qué tal se movieron sus dedos de la mano **izquierda**? | 1 | 2 | 3 | 4 | 5 |
| 3. ¿Qué tal se movió su muñeca **izquierda**? | 1 | 2 | 3 | 4 | 5 |
| 4. ¿Cómo fue la fuerza en su mano **izquierda**? | 1 | 2 | 3 | 4 | 5 |
| 5. ¿Cómo describiría el tacto de su mano **izquierda**? | 1 | 2 | 3 | 4 | 5 |

1. Las siguientes preguntas se refieren la capacidad de su(s) mano(s) para realizar ciertas tareas **durante la semana pasada** (Por favor, rodee con un círculo 1 respuesta para cada pregunta).
   1. ¿Cuánta dificultad tuvo para realizar las siguientes actividades usando su mano **derecha**?

|  | Ninguna | Poca | Alguna | Moderada | Mucha |
| --- | --- | --- | --- | --- | --- |
| 1. Girar el pomo de una puerta | 1 | 2 | 3 | 4 | 5 |
| 2. Recoger una moneda | 1 | 2 | 3 | 4 | 5 |
| 3. Sujetar un vaso de agua | 1 | 2 | 3 | 4 | 5 |
| 4. Girar la llave en una cerradura | 1 | 2 | 3 | 4 | 5 |
| 5. Sujetar una sartén | 1 | 2 | 3 | 4 | 5 |

- 1. ¿Cuánta dificultad tuvo para realizar las siguientes actividades usando su mano **izquierda**?

|  | Ninguna | Poca | Alguna | Moderada | Mucha |
| --- | --- | --- | --- | --- | --- |
| 1. Girar el pomo de una puerta | 1 | 2 | 3 | 4 | 5 |
| 2. Recoger una moneda | 1 | 2 | 3 | 4 | 5 |
| 3. Sujetar un vaso de agua | 1 | 2 | 3 | 4 | 5 |
| 4. Girar la llave en una cerradura | 1 | 2 | 3 | 4 | 5 |
| 5. Sujetar una sartén | 1 | 2 | 3 | 4 | 5 |

- 1. ¿Cuánta dificultad tuvo para realizar las siguientes actividades usando **ambas manos**?

|  | Ninguna | Poca | Alguna | Moderada | Mucha |
| --- | --- | --- | --- | --- | --- |
| 1. Abrir un tarro | 1 | 2 | 3 | 4 | 5 |
| 2. Abotonar una camisa/blusa | 1 | 2 | 3 | 4 | 5 |
| 3. Comer con cuchillo y tenedor | 1 | 2 | 3 | 4 | 5 |
| 4. Llevar una bolsa de la compra | 1 | 2 | 3 | 4 | 5 |
| 5. Lavar los platos | 1 | 2 | 3 | 4 | 5 |
| 6. Lavarse el pelo | 1 | 2 | 3 | 4 | 5 |
| 7. Atar los cordones/anudar | 1 | 2 | 3 | 4 | 5 |

1. Las siguientes preguntas se refieren a su capacidad a la hora de realizar su **trabajo habitual** (incluyendo

tanto las tareas domésticas como las educativas) durante las **últimas 4 semanas** (Por favor, rodee con un círculo 1 respuesta para cada pregunta).

|  | Ninguna | Poca | Alguna | Moderada | Mucha |
| --- | --- | --- | --- | --- | --- |
| 1. ¿Con qué frecuencia le fue imposible hacer su trabajo  por los problemas derivados de su(s) mano(s)/muñeca(s)? | 1 | 2 | 3 | 4 | 5 |
| 2. ¿Con qué frecuencia tuvo que reducir su jornada de trabajo  diario por los problemas derivados de su(s) mano(s)/muñeca(s)? | 1 | 2 | 3 | 4 | 5 |
| 3. ¿Con qué frecuencia tuvo que reducir su ritmo de trabajo por  los problemas derivados de su(s) mano(s)/muñeca(s)? | 1 | 2 | 3 | 4 | 5 |
| 4. ¿Con qué frecuencia se vieron mermados sus logros por los problemas derivados de su(s) mano(s)/muñeca(s)? | 1 | 2 | 3 | 4 | 5 |
| 5. ¿Con qué frecuencia tardó más en realizar las tareas en su  trabajo por los problemas derivados de su(s) mano(s)/muñeca(s)? | 1 | 2 | 3 | 4 | 5 |

1. Las siguientes preguntas se refieren al grado de **dolor** que experimentó en su(s) mano(s)/ muñeca(s)

**durante la semana pasada** (Por favor, rodee con un círculo 1 respuesta por cada pregunta).

1. Las siguientes preguntas se refieren a su mano **derecha**
   1. ¿Con qué frecuencia tuvo dolor en su mano/muñeca **derecha**? 1.Siempre
   2. Frecuentemente
   3. A veces 4.Rara vez 5.Nunca

Si usted respondió **nunca** a la **pregunta IV-1**, por favor evite contestar las siguientes preguntas y pase al siguiente apartado.

1. Por favor, describa el dolor que tiene en su mano/muñeca **derecha**
   1. Muy suave 2.Suave 3.Moderado 4.Severo 5.Muy severo

|  | Siempre | Frecuente  -mente | A  veces | Raramente | Nunca |
| --- | --- | --- | --- | --- | --- |
| 3. ¿Con qué frecuencia el dolor en su mano/muñeca  **derecha** provocó alteraciones en su sueño? | 1 | 2 | 3 | 4 | 5 |
| 4. ¿Con qué frecuencia el dolor en su mano/muñeca  **derecha** interfirió en sus actividades diarias (tales como comer o bañarse)? | 1 | 2 | 3 | 4 | 5 |
| 5. ¿Con qué frecuencia el dolor en su mano /muñeca  **derecha** afectó de manera negativa a su estado de ánimo? | 1 | 2 | 3 | 4 | 5 |

1. Las siguientes preguntas se refieren a su mano **izquierda**
   1. ¿Con qué frecuencia tuvo dolor en su mano/muñeca **izquierda**? 1.Siempre
   2. Frecuentemente

3.A veces 4.Rara vez 5.Nunca

Si usted respondió **nunca** a la **pregunta IV-1**, por favor evite contestar las siguientes preguntas y pase al siguiente apartado.

1. Por favor, describa el dolor que tiene en su mano/muñeca **izquierda**
   1. Muy suave 2.Suave 3.Moderado 4.Severo 5.Muy severo

|  | Siempre | Frecuente  -mente | A  veces | Raramente | Nunca |
| --- | --- | --- | --- | --- | --- |
| 3. ¿Con qué frecuencia el dolor en su mano/muñeca  **izquierda** provocó alteraciones en su sueño? | 1 | 2 | 3 | 4 | 5 |
| 4. ¿Con qué frecuencia el dolor en su mano/muñeca  **izquierda** interfirió en sus actividades diarias (tales como o bañarse)? | 1 | 2 | 3 | 4 | 5 |
| 5. ¿Con qué frecuencia el dolor en su mano /muñeca  **izquierda** afectó de manera negativa a su estado de ánimo? | 1 | 2 | 3 | 4 | 5 |

V.A. Las siguientes preguntas se refieren a la apariencia de su mano **derecha** durante la semana pasada (Por favor, rodee con un círculo 1 respuesta para cada pregunta).

| Totalmente  de acuerdo | | De  acuerdo | Indife -  rente | En  desacuerdo | Totalmente en  desacuerdo |
| --- | --- | --- | --- | --- | --- |
| 1.Estuve satisfecho/a con la apariencia de mi mano  **derecha** | 1 | 2 | 3 | 4 | 5 |
| 2. La apariencia de mi mano **derecha** a veces me hizo  sentir incómodo/a en público | 1 | 2 | 3 | 4 | 5 |
| 3. La apariencia de mi mano **derecha** me deprimió | 1 | 2 | 3 | 4 | 5 |
| 4. La apariencia de mi mano **derecha** interfirió con mis actividades sociales habituales | 1 | 2 | 3 | 4 | 5 |

B. Las siguientes preguntas se refieren a la apariencia de su mano **izquierda** durante la semana pasada (Por favor, rodee con un círculo 1 respuesta para cada pregunta).

| Totalmente de acuerdo | | De acuerdo | Indife- rente | En desacuerdo | Totalmente en desacuerdo |
| --- | --- | --- | --- | --- | --- |
| 1.Estuve satisfecho/a con la apariencia de mi mano  **izquierda** | 1 | 2 | 3 | 4 | 5 |
| 2.La apariencia de mi mano **izquierda** a veces me hizo  sentir incómodo/a en público | 1 | 2 | 3 | 4 | 5 |
| 3.La apariencia de mi mano **izquierda** me deprimió | 1 | 2 | 3 | 4 | 5 |
| 4.La apariencia de mi mano **izquierda** interfirió con  mis actividades sociales habituales | 1 | 2 | 3 | 4 | 5 |

VI.A. Las siguientes preguntas se refieren al grado de satisfacción con su mano/muñeca **derecha** durante la semana pasada (Por favor, rodee con un círculo 1 respuesta para cada pregunta).

|  | Muy  Satisfecho/a | Algo  Satisfecho/a | Indife-  rente | Algo  Insatisfecho/a | Muy  Insatisfecho/a |
| --- | --- | --- | --- | --- | --- |
| 1. En general, con la función de su mano **derecha** | 1 | 2 | 3 | 4 | 5 |
| 2.Con la movilidad de los dedos en su mano **derecha** | 1 | 2 | 3 | 4 | 5 |
| 3. Con la movilidad de su muñeca **derecha** | 1 | 2 | 3 | 4 | 5 |
| 4. Con la fuerza de su mano **derecha** | 1 | 2 | 3 | 4 | 5 |
| 5. Con el nivel de dolor de su mano **derecha** | 1 | 2 | 3 | 4 | 5 |
| 6. Con el tacto de su mano **derecha** | 1 | 2 | 3 | 4 | 5 |

B. Las siguientes preguntas se refieren al grado de satisfacción con su mano/muñeca **izquierda** durante la semana pasada (Por favor, rodee con un círculo 1 respuesta para cada pregunta).

|  | Muy satisfecho/a | Algo satisfecho/a | Indife- rente | Algo insatisfecho/a | Muy insatisfecho/a |
| --- | --- | --- | --- | --- | --- |
| 1. En general, con la función de su mano **izquierda** | 1 | 2 | 3 | 4 | 5 |
| 2.Con la movilidad de los dedos en su mano  **izquierda** | 1 | 2 | 3 | 4 | 5 |
| 3. Con la movilidad de su muñeca **izquierda** | 1 | 2 | 3 | 4 | 5 |
| 4. Con la fuerza de su mano **izquierda** | 1 | 2 | 3 | 4 | 5 |
| 5. Con el nivel de dolor de su mano **izquierda** | 1 | 2 | 3 | 4 | 5 |
| 6. Con el tacto de su mano **izquierda** | 1 | 2 | 3 | 4 | 5 |

Por favor, añada la siguiente información sobre usted mismo (Por favor, rodeé con un círculo 1 respuesta para cada pregunta).

1. ¿Es usted diestro/a o zurdo/a?
   1. Diestro/a
   2. Zurdo/a
   3. Ambidiestro/a
2. ¿Qué mano le da más problemas?
   1. Derecha
   2. Izquierda
   3. Ambas
3. ¿Ha cambiado de trabajo desde que tuvo problemas con sus manos?
   1. Si
   2. No

Por favor describa el tipo de trabajo que hacía **antes** de tener la lesión en su(s) mano(s)

Por favor describa el tipo de trabajo que está haciendo **ahora**

1. ¿Cuál es su género?
   1. Hombre
   2. Mujer
2. ¿Cuál es su origen étnico?
   1. Europeo/a
   2. Africano/a
   3. Hispano/a
   4. Asiático/a -Islas del Pacífico
3. ¿Cuál es el nivel más alto de estudios que usted recibió?
   1. Sin estudios/ Menor al Graduado escolar
   2. Graduado escolar
   3. Estudios universitarios sin finalizar
   4. Grado universitario
   5. Posgrado
4. ¿Cuáles son sus ingresos familiares aproximados, incluyendo salario, subsidio de discapacidad, pensión de jubilación y ayudas sociales?

a. <10.000€

b. 10.000 a 19.999 €

c. 20.000 a 29.999€

d. 30.000 a 39.999€

e. 40.000 a 49.999€

f. 50.000 a 59.999 €

g. 60.000 a 69.999€. h. >70.000€

1. ¿Está su lesión cubierta por un seguro de accidentes de trabajo?
   1. Si
   2. No
